# What Have In Vitro Co-Culture Models Taught Us about the Contribution of Epithelial-Mesenchymal Interactions to Airway Inflammation and Remodeling in Asthma?

**DOI:** 10.3390/cells9071694

**Published:** 2020-07-15

**Authors:** Emmanuel Twumasi Osei, Steven Booth, Tillie-Louise Hackett

**Affiliations:** 1 Centre for Heart Lung Innovation, St. Paul’s Hospital, Vancouver, BC V6Z 1Y6, Canada; Steve.Booth@hli.ubc.ca (S.B.); Tillie.Hackett@hli.ubc.ca (T.-L.H.); 2 Department of Anesthesiology, Pharmacology, and Therapeutics, University of British Columbia, Vancouver, BC V6T 1Z3, Canada

**Keywords:** asthma, in vitro co-culture models, epithelial-mesenchymal trophic unit, airway epithelial cells, lung fibroblasts, airway smooth muscle cells, cross-talk

## Abstract

As the lung develops, epithelial-mesenchymal crosstalk is essential for the developmental processes that drive cell proliferation, differentiation, and extracellular matrix (ECM) production within the lung epithelial-mesenchymal trophic unit (EMTU). In asthma, a number of the lung EMTU developmental signals have been associated with airway inflammation and remodeling, which has led to the hypothesis that aberrant activation of the asthmatic EMTU may lead to disease pathogenesis. Monoculture studies have aided in the understanding of the altered phenotype of airway epithelial and mesenchymal cells and their contribution to the pathogenesis of asthma. However, 3-dimensional (3D) co-culture models are needed to enable the study of epithelial-mesenchymal crosstalk in the setting of the in vivo environment. In this review, we summarize studies using 3D co-culture models to assess how defective epithelial-mesenchymal communication contributes to chronic airway inflammation and remodeling within the asthmatic EMTU.

## 1. Introduction

Asthma is defined as a heterogeneous disease characterized by chronic airway inflammation and variable airways hyperresponsiveness [1]. The global prevalence of asthma is expected to increase to approximately 400 million people by 2025 [1]. Although current treatments can help alleviate symptoms, there is still no cure for asthma, resulting in 346,000 early deaths each year worldwide [2]. Many phase II clinical trials for complex diseases like asthma have failed due to the lack of human disease models for preclinical validation. Human in vitro models are needed to investigate the underlying pathogenic mechanisms of airway inflammation and remodeling with the goal of identifying therapeutic targets for asthma. 

**Figure 1:**
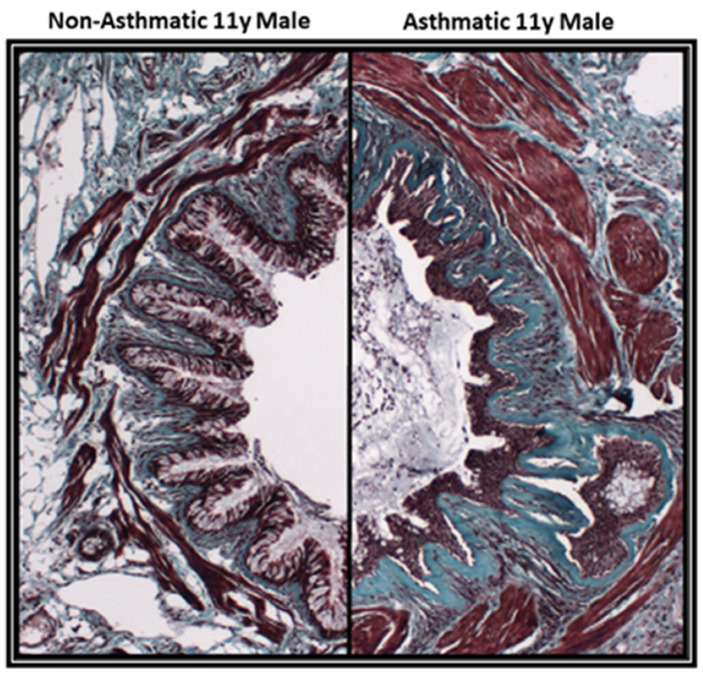
Features of airway remodeling in the large airways in asthma. Airway sections from formalin-fixed paraffin-embedded (FFPE) tissue stained with Masson’s trichrome stain for collagen (blue-green), cytoplasm and intercellular space (light purple) and keratin and muscle (red). The left image in the panel: a large airway from a normal control individual with no respiratory disease. The right image in the panel: an age- and sex-matched large airway of an asthmatic individual showing airway remodeling including (i) increased smooth muscle mass; (ii) damaged airway epithelium; (iii) basement membrane thickening (iv) mucus plugging of the airway lumen and (v) subepithelial fibrosis.

The inflammatory profile of asthmatic patients is very heterogeneous. For patients with allergic asthma, antigen-specific IgE cross-linking of mast cell-surface Fc receptors leads to degranulation and the release of bronchoconstrictors (e.g., histamine and leukotrienes) that cause airways hyperresponsiveness (AHR) [3,4,5,6]. Allergen-exposure also results in chronic airway inflammation due to airway epithelial-derived release of inflammatory mediators leading to T helper 2 (T_H_2)-inflammation and eosinophilia [5]. In corticosteroid-resistant asthma, chronic inflammation is associated with predominantly neutrophilic-derived mediators [6]. In nonallergic asthma that accounts for 10-33% of patients, they have normal serum IgE levels, and the chronic airway inflammation present consists of increased numbers of neutrophils and activation of the IL-17 pathway [7,8]. In addition to airways hyperresponsiveness, patients may also experience fixed airflow obstruction due to airway remodeling [9]. Histopathological studies of airway remodeling in bronchial biopsies and explanted lung-tissues have shown alterations in airway epithelium (loss of epithelial barrier function, goblet cell hyperplasia), the mesenchyme (airway smooth muscle (ASM) hypertrophy and hyperplasia, increased numbers of myofibroblasts, and angiogenesis) and extracellular matrix (ECM) (thickening of the basement membrane and subepithelial fibrosis) [2,10,11,12] (Figure 1). 

It has been proposed that an abnormal communication between the airway epithelium and the lung mesenchyme may play an important role in driving airway inflammation and remodeling in asthma [2,13,14]. This communication mirrors the reciprocal, temporal, spatial and cell-type-specific interactions between the endoderm and mesoderm essential for driving branching morphogenesis during lung development. In 1999, Plopper and Evans introduced the concept of the epithelial-mesenchymal trophic unit (EMTU) and the fact that communication of cells within the EMTU plays a major role in lung development, repair, and homeostasis [15]. Since then, many studies have focused on how an abnormal reactivation of the EMTU in response to chronic mucosal injury may play a role in airway inflammation and remodeling in asthma [2,13]. 

Although monoculture studies have enabled us to understand the abnormal phenotype of the airway epithelium and mesenchymal cells in asthma, 3D in vitro co-culture models have enabled the assessment of interactions within the multicellular EMTU environment. In this review, we provide a summary of studies that have used 3D in vitro models to understand the interaction of the epithelium with the mesenchyme in the asthmatic EMTU and how they contribute to airway inflammation and remodeling (cell proliferation and ECM homeostasis) in asthma.

## 2. Co-Culture Models Used to Assess Epithelial-Mesenchymal Crosstalk 

Within the lungs, the epithelium is specialized along the airway tree ranging from a pseudostratified epithelium consisting of basal, ciliated and goblet cells within the large conducting airways, club cells within the small airways and a simple squamous epithelium formed of alveolar type I and II cells within alveoli, that all attach via an extracellular matrix (ECM) basement membrane [16]. Beneath the basement membrane, the various mesenchymal cells range from airway (myo)fibroblasts, endothelial cells, and airway smooth muscle (ASM) cells within the lamina propria of the large conducting airways, to the parenchymal fibroblasts within the interstitium of parenchymal tissue [17]. A major component of the EMTU is the ECM, which is made up of an intricate network of macromolecular proteins including collagens, fibronectin, and various proteoglycans [18]. The ECM is essential for tissue structure and the programming of cell migration, proliferation, viability, and morphology [18]. 

The approach of using co-culture systems is to model the normal physical cell environment and the complex interactions between different cell types and their ECM within the EMTU. The co-culture models used to assess cellular communication in the lung EMTU have included conditioned medium-exposure experiments, epithelial-mesenchymal co-cultures, and 3D-ECM (co-culture) models. These models have been established both with lung epithelial and mesenchymal cell lines [19] and primary cells isolated either from animal models [20] or human asthmatic and control lungs [21]. 

**Figure 2:**
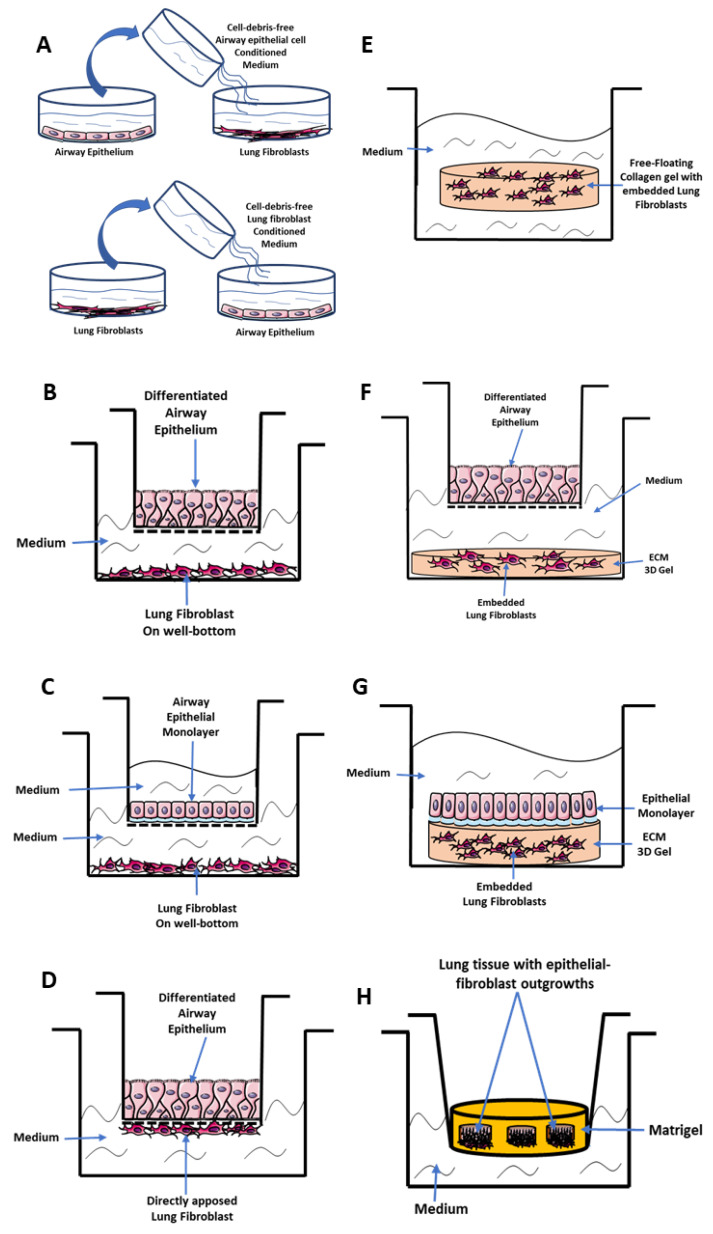
In vitro co-culture models of epithelial-mesenchymal crosstalk in asthma. (**A**) Conditioned medium exposure models where cell-debris free medium from cultured epithelial cells is placed on fibroblasts and vice versa to assess soluble mediators involved in cellular crosstalk. (**B**,**C**) Indirect co-culture systems showing (**B**) airway epithelium differentiated at air-liquid interface and (**C**) airway epithelial cell monolayer grown in submerged medium on transwell inserts which are placed in culture wells with fibroblasts grown at the bottom. (**D**) A direct co-culture system with differentiated airway epithelial cells in a transwell with co-cultured lung fibroblasts directly underneath the transwell. (**E**) A free-floating 3D collagen gel in which lung fibroblasts are embedded. (**F**–**H**) 3D ECM co-culture systems with fibroblasts embedded in extracellular matrix (ECM) gels co-cultured (**F**) indirectly with differentiated airway epithelium grown in a transwell or (**G**) directly with an epithelial monolayer grown on the gel. (**H**) A special 3D ECM where fragments of lung tissue explants are placed in matrigel to allow for epithelial-fibroblast outgrowths.

In conditioned medium-exposure experiments, cells are cultured separately in monolayers after which cell-debris-free culture medium from one cell type is harvested and incubated with the other cell type (Figure 2A). This enables the assessment of cell-phenotype and how soluble mediators released from one cell-type may affect the phenotype of another. In co-culture models, primary airway epithelial cells (PAECs) can be differentiated at an air-liquid interface (ALI) (Figure 2B), or maintained in a monolayer (Figure 2C) in transwell inserts that have semipermeable membranes, and placed in co-culture with mesenchymal cells. In the indirect co-culture model, the transwell with PAECs is suspended over mesenchymal cells cultured at the bottom of the well (Figure 2B,C). In the direct co-culture model, mesenchymal cells are cultured directly underneath the transwell insert with PAECs on top to help assess cell-cell contact and interactions [22,23] (Figure 2D). 

To understand the 3D ECM environment within the EMTU, mesenchymal cells can be embedded in free-floating low-tension ECM gels to determine how they repair and synthesize ECM proteins (Figure 2E). Other variations involve an ECM co-culture model that entails embedding mesenchymal cells in ECM protein gels (e.g., Matrigel or collagen I gels) and co-culturing them with PAECs either in a transwell (Figure 2F) or cultured directly on the gel surface (Figure 2G). In another variation, lung tissue explants can be embedded in Matrigel, which causes multilayered epithelial-fibroblast cell-outgrowths to form in close contact, supported by a 3D ECM environment (Figure 2H) [24]. 

The various co-culture models allow for cellular communication through the release of soluble mediators from both epithelial and mesenchymal cells which mimic in vivo conditions in the airways. In addition, 3D ECM co-culture models enable the assessment of cell-ECM interactions. An important point to consider for the various models is the need for careful assessment of the media used to ascertain the viability of the different cells being co-cultured. Another caveat is the ability to isolate RNA and protein to enable the assessment of cellular crosstalk as the different models increase in complexity. In the sections below, we review studies that have used in vitro 3D co-culture models to assess how epithelial-mesenchymal crosstalk plays a role in airway inflammation and remodeling in asthma.

## 3. Asthma EMTU Models Assessing Airway Inflammation 

In vivo, many airway inflammatory mediators are increased in asthmatic patients including thymic stromal lymphopoietin (TSLP), eotaxin-1 and granulocyte-monocyte colony-stimulating factor (GM-CSF), which have been shown to drive eosinophilia and T_H_2-inflammation (interleukin (IL)-4, IL-5, IL-9, IL-13), whereas in neutrophilic asthma, IL-8, IL-6, IL-1, and tumor necrosis factor (TNF)-α have been shown to drive chronic airway inflammation [6]. The use of co-culture systems has allowed for the assessment of which specific cytokines drive abnormal epithelial-mesenchymal crosstalk in the asthmatic-EMTU.

An increased ASM cell mass and hypercontractility are important causes of AHR in asthmatics. However, ASM cells can also contribute to airway inflammation in the asthmatic-EMTU [25]. Allard et al. stimulated non-asthma-derived PAEC-ALI’s with human rhinovirus (HRV) to simulate viral exacerbations and co-cultured these with ASM cells derived from healthy controls or asthmatic patients [26]. Following HRV infection of PAEC-ALI’s, the co-culture of asthmatic-derived ASM cells caused the release of chemokines (chemokine (C-C) motif ligand (CCL)2, CCL5, CCL17, chemokine (C-X-C) motif ligand (CXCL)1, CXCL2, CXCL5, CXCL6, and CXCL9), the cytokines IL-1α, IL-6 and TNF-α by PAECs, but not when co-cultured with control-derived ASM cells. Further, when the conditioned media from the HRV infected PAEC-ALI and asthmatic-derived ASM co-cultures was used in Boyden chamber experiments, the release of these cytokines, and in particular CCL5, led to the increased migration of monocytes, which did not occur with the uninfected PAEC-ALI and control-derived ASM co-cultures [26]. This co-culture study highlighted the complexity of epithelial-mesenchymal crosstalk in driving inflammatory cell recruitment in asthma.

To understand the role of fibrocyte infiltration into the airway smooth muscle of asthmatic patients [27], Lin et al., co-cultured monocyte-derived circulating progenitor-cells (fibrocytes) with ASM cells. In these co-culture experiments, fibrocytes did not affect ASM-proliferation or the expression of transforming growth factor (TGF)-β1, α-smooth muscle actin (SMA) or myosin light chain kinase, but they did cause an increased release of IL-8 and IL-6 from ASM cells [28]. Through mechanistic experiments with siRNA silencing, it was found that the increase in IL-8 and IL-6-release was due to the activation of transcription factors NF-κB-p65 and extracellular signal-regulated kinase (ERK)1/2 in ASM cells [28]. This study demonstrated that ASM crosstalk with progenitor fibrocytes may play an important role in driving monocyte and neutrophil influx in the asthmatic EMTU. 

In several epithelial-fibroblast co-culture experiments, the epithelial-release of IL-1α has been shown to control the phenotype of fibroblasts within the normal lung EMTU [29,30,31]. Osei and colleagues recently assessed the kinetics of IL-1-release from differentiating PAECs derived from asthmatics and healthy controls and found greater IL-1α expression and release in asthmatic-PAECs when in a monolayer (before differentiation) compared to control-PAECs [31]. In support of these data, IL-1 has been shown to increase in broncho-alveolar lavage (BAL), sputum and lung tissue in asthmatics which causes T_H_2-inflammation, eosinophilia and AHR [32,33]. To further assess the effects of IL-1 in the asthmatic EMTU, Osei et al., stimulated both asthmatic and control-derived airway fibroblasts with IL-1α/IL-1β which caused an increased release of fibroblast-derived TSLP, GM-CSF, IL-8 and IL-6 with no differences in asthmatic and non-asthmatic-derived fibroblasts [31]. TSLP and GM-CSF stimulate increased eosinophil and T_H_2-inflammation, while IL-8 and IL-6 are important for the influx and maintenance of neutrophils in the airways [6]. This study highlighted how increased IL-1α release from the asthmatic epithelium could contribute to airway inflammation through fibroblast-mediated inflammatory responses. 

In addition to epithelial-derived mediators driving mesenchymal airway inflammation, cross-talk in the EMTU may also play a role in suppressing mesenchymal cell activation [34]. Calzetta et al. studied the effects of brain natriuretic peptide (BNP), a cardiac and vascular-derived hormone which causes bronchorelaxation in asthmatics, on the asthmatic EMTU [34]. When the airway epithelial BEAS-2B cell line was treated with BNP, the epithelial-derived release of acetylcholine into the conditioned media caused an increased expression of inducible nitric oxide synthase (iNOS) and myosin phosphatase target subunit-1 (MYPT1) in asthmatic-derived ASM cells compared to control ASM cells [34]. The increased expression of iNOS and MYPT1 in asthmatic-derived ASM cells led to a decrease in histamine sensitivity leading to reduced hypercontractility, which was not observed in control ASM cells. Importantly, this protective effect of BNP reducing asthmatic ASM cell hypercontractility to histamine was shown not to occur with the direct culture of BNP alone, but required epithelial-derived acetylcholine release, demonstrating the importance of understanding epithelial-mesenchymal cell cross-talk when studying asthma disease mechanisms [34].

Human mesenchymal stem cells (hMSCs) have been shown to play a role in modulating lung inflammation in different disease settings [35]. Hong and colleagues stimulated the airway epithelial BEAS-2B cell line with polyinosinic:polycytidylic acid (polyI:C) to simulate viral exposure in the airways of severe steroid-resistant asthmatics and co-cultured these with hMSCs. PolyI:C stimulation led to increased IL-8 release from BEAS-2B cells. However, in co-culture, hMSCs significantly reduced the increased production of IL-8 from polyI:C-stimulated BEAS-2B cells [35]. In addition, when isolated human peripheral blood mononuclear cells (PBMCs) were stimulated with CD28, a T-cell activator, and co-cultured with hMSCs, there was a reduction of the concentrations of interferon-γ and the T_H_2 cytokine IL-4 released from PBMCs [35]. This study showed that hMSC and PAEC crosstalk may be an important mechanism for the immune response against viral-induced exacerbations in asthma.

Kuo et al. investigated the role of hMSCs on PAEC-derived inflammation during bronchoconstriction [36]. The authors demonstrated that hyperstretching of PAECs to mimic increased mechanical strain in asthmatic airways led to increased release of the inflammatory cytokines IL-1α and IL-8 due to high expression of miR-155. The authors found that increased miR-155 targets and suppresses the production of Src homology 2 domain-containing inositol 5-phosphatase 1 (SHIP1), which leads to a subsequent JNK signaling activation. The co-culture of hMSCs with hyperstretched PAECs led to the downregulation of IL-8 due to an increase in the anti-inflammatory cytokine IL-10 that downregulates miR-155 expression [36]. These studies demonstrated that crosstalk between hMSCs and the airway epithelium may serve to control excessive inflammation in asthmatic airways.

Taken together, these studies (summarized in Table 1) showed how in vitro co-culture models of the asthmatic EMTU can be used to identify mechanisms of airway inflammation. In the asthmatic EMTU, the epithelial release of inflammatory cytokines (CCL-5, IL-1α, TNF-α, IL-8, and IL-6) can regulate mesenchymal inflammation to cause eosinophilia and T_H_2 inflammation (TSLP, GM-CSF) as well as neutrophilic inflammation (TNF-α, IL-8, IL-6). These studies also highlighted the complexity of crosstalk within the EMTU and how hMSCs and mediators such as BNP may serve as novel therapeutic alternatives for treating neutrophilic inflammation in severe asthma.

## 4. Asthma EMTU Models Assessing Airway Remodeling

### 4.1. Cellular Proliferation 

The abnormal proliferation of epithelial and mesenchymal cells is an important feature of airway remodeling in asthma [37,38]. In a study to assess the epithelial-mediators important for fibroblast-proliferation in asthma, Zhang et al., developed a 3D-ECM co-culture model where myofibroblasts isolated from the airway wall were embedded in collagen I gels with the airway epithelial 16HBE14o- cell-line co-cultured on the gel surface [19]. Co-cultured 16HBE14o- cells were then treated with poly-L-arginine to mimic eosinophil granule cationic protein release in allergic asthmatics, which increased the proliferation rate of the co-cultured myofibroblasts. The increased myofibroblast proliferation was due to greater levels of airway epithelial-derived growth factors including endothelin (ET)-1, platelet-derived growth factor (PDGF), insulin-like growth factor (IGF)-1 and TGF-β2 release following poly-L-arginine stimulation [19]. In support of these findings, ET-1, PDGF, IGF-1 and TGF-β2 have been shown to increase in BAL fluid of asthmatic patients [39,40,41]. This study showed that the epithelial release of fibrogenic growth factors after airway damage caused increased myofibroblast proliferation, which is important as this has been demonstrated in the airways of asthmatics and shown to strongly correlate with airway fibrosis [42]. 

With regards to airway smooth muscle, Malavia et al. used a differentiated PAEC-ASM co-culture model to demonstrate that epithelial injury through repeated scrape wounds caused an increased release of matrix metalloproteinase (MMP)-9, IL-8 and IL-6 from PAECs that stimulated increased ASM proliferation [43]. Of these mediators, it was discovered through the siRNA knockdown of specific genes, that MMP-9 was the main mediator driving ASM-proliferation, possibly through ERK1/2 and the mitogen protein kinase (MAPK) pathways [43]. MMP-9 is increased in BAL and sputum of asthmatics compared to control individuals and correlated with airway fibrosis [44]. Although MMPs are proteolytic enzymes involved in matrix turnover [44,45], this study demonstrated that the release of MMP-9 in the asthmatic-EMTU also had mitogenic properties on ASM cells.

To understand the effects of bronchoconstriction on the EMTU, Lan and colleagues showed that compression of PAECs led to increased production of ET-1 [46]. Conditioned-medium from the compressed PAECs led to an ET-1-mediated proliferation of ASM cells after exposure experiments [46]. ET-1 release from compressed PAECs also augmented ASM contractile response to histamine challenge. This study demonstrated that different forms of epithelial injury in the asthmatic airways, such as compression due to bronchoconstriction, may cause epithelial-driven ASM proliferation through the release of growth factors such as ET-1. This epithelial-mesenchymal cross-talk may provide a mechanism for the increased smooth muscle mass within asthmatic airways [47].

In other studies to assess how mesenchymal cells regulate epithelial proliferation, Semlali et al., established a 3D co-culture model using a collagen scaffold in which airway fibroblasts from mild asthmatics and non-asthmatics were embedded and co-cultured with PAECs from the same individuals grown at ALI on top of the scaffold [48]. They found that co-cultures of mild asthmatic PAECs and airway fibroblasts had decreased PAEC proliferation rates compared to non-asthmatic PAEC-fibroblast co-cultures. This defect was due to abnormal phosphorylation of epidermal growth factor receptor (EGFR) in the mild asthmatic-airway epithelium due to increased release of the activated form of TGF-β1 by asthma-derived airway fibroblasts [48]. In line with this, TGF-β1 and EGFR have been shown to have increased expression in asthmatic individuals and correlate with thickening of the airway wall [49] pointing to the importance of epithelial-mesenchymal communication in asthmatic airway remodeling. 

In contrast, Haj-Salem and colleagues found increased proliferation rates of PAECs when cultured with conditioned media from severe asthmatic-derived airway fibroblasts compared to nonasthmatic airway fibroblasts. They found that exosomes released from severe-asthmatic-derived airway fibroblasts had a low expression of TGF-β2 and high expression of cytokines IL-6, IL-8, CCL-1, and GRO-α. Through gain of function assays, it was found that the low levels of TGF-β2 in asthmatic-derived airway fibroblasts caused the increased PAEC-proliferation [50]. Comparing the findings of this study to that of Semlali et al., [48] shows how differences in the expression levels of different TGF-β isoforms in airway fibroblasts from mild or severe asthmatics may drive decreased or increased PAEC-proliferation. A decrease in PAEC proliferation rates due to decreased airway fibroblast exosome-TGF-β2 expression may point to an impaired cellular ability to effectively repair damage within the airways of asthmatics. 

In summary, these studies (summarized in Table 2) highlighted how crucial epithelial-mesenchymal crosstalk is for cellular proliferation which is an important feature of airway remodeling in asthma. In co-culture experiments, the epithelial-release of inflammatory mediators (IL-8, IL-6), proteolytic enzymes (MMP-9) and growth factors (TGF-β, ET-1, PDGF, and IGF-1) drive increased fibroblast and ASM proliferation in asthmatic airways. On the other hand, airway fibroblasts in asthmatics may stimulate an increase or decrease in PAEC-proliferation through the differential expression of different TGF-β isoforms in different asthmatic phenotypes.

### 4.2. Extracellular Matrix Homeostasis 

In asthma, the increased production of ECM proteins by elevated numbers of mesenchymal cells contributes to airway wall fibrosis [15,51,52,53]. To determine the specific effects of epithelial-fibrogenic-factor release on fibroblast-ECM production, Thompson and colleagues mimicked asthmatic airway epithelial injury with a scrape wound on PAECs co-cultured on a 3D collagen I gel embedded with lung fibroblasts. PAEC injury caused the release of active TGF-β2 that caused an increased expression of α-SMA, tenascin-C and fibrillar collagen in the collagen-embedded lung fibroblasts [54]. In another study, Morishima and colleagues developed an EMTU model with an ALI of guinea pig PAECs grown on the apical side of a human amnion chamber which was co-cultured on top of airway fibroblasts seeded on plastic sheets in 6-well culture-plates [55]. A scrape wound made on the PAECs to mimic epithelial damage in asthmatics was completely repaired by the eighth day after wounding, which mimicked the delayed wound repair in asthmatic airways [56]. During the transient period of wound repair, PAECs caused myofibroblast-induction and increased collagen I expression in airway fibroblasts via the epithelial-release of TGF-β1 and thrombospondin (TSP) [55]. Since α-SMA expressing myofibroblasts increased in asthmatic airways together with increased TGF-β, TSP and collagen expression [18,57], these studies point to the direct involvement of epithelial-mesenchymal crosstalk in increased ECM production in the asthmatic airways. 

To assess disease-specific differences in ECM production due to EMTU crosstalk, Reeves et al., performed co-culture studies with PAECs and fibroblasts isolated from asthmatic patients and control individuals. Their co-culture models showed that compared to non-asthmatics, asthmatic-derived PAEC-ALIs caused increased expression of α-SMA and collagen 1 [58], and the nonsulfated glycosaminoglycan hyaluronan (HA) [59] in control lung fibroblasts, which the authors concluded may indicate fibroblast-to-myofibroblast (FMT) transition. The increase of fibroblast collagen 1 and α-SMA expression were due to increased production of the inflammatory cytokine prostaglandin (PG)E_2_ [58], while increased HA expression was due to a higher production of the enzymes hyaluronan synthase (HSA) 2 and 3 [59] in asthmatic compared to non-asthmatic PAEC-ALIs. The secreted HA from asthma-derived PAEC-ALI co-cultures was shown to promote the binding of leukocytes [59]. Further, the induced expression of α-SMA and collagen 1 in control lung fibroblasts upon co-culture with asthma-PAEC-ALIs was negatively correlated with lung function [58]. These studies showed that an increased ECM deposition in the airways of asthmatic patients correlates with poor lung function and may be due to increased production of epithelial-inflammatory mediators (PGE_2_) and fibrogenic enzymes (HSA2/3) that drive myofibroblast transition in the airways. 

Hastie et al. assessed the effects of allergen exposure in the asthmatic-EMTU [60]. The authors challenged asthmatic and non-asthmatic subjects with Ragweed allergen before collecting PAECs via bronchial brushing and BAL fluid from the patients. BAL fluid from the asthmatics had an increased number of eosinophils compared to non-asthmatics. Asthmatic and non-asthmatic-PAECs were then co-cultured for 24 h with their corresponding BAL fluids [60]. Conditioned medium collected from the asthmatic PAEC-BAL co-cultures stimulated the expression of collagen III in normal lung fibroblasts compared to non-asthmatic PAEC-BAL co-cultures [60]. Increased TGF-β1 production from asthmatic PAECs was shown to cause increased collagen III expression in lung fibroblasts [60]. The results of this study determined that allergen exposure in asthmatic airways damaged PAECs and caused an increased TGF-β1 release to drive collagen III production by lung fibroblasts in the asthmatic EMTU.

In the normal breathing lung, various cells are exposed to varying degrees of mechanical strain, which is highly increased in asthma as a result of bronchoconstriction [61]. Swartz and colleagues showed in a co-culture model that varying degrees of mechanical stress on PAEC-ALIs caused lung fibroblasts to increase their ECM (collagen III, fibronectin) and MMP-9 production while decreasing tissue inhibitor of metalloproteinases (TIMP)1 expression [62]. In a separate study, Tschumperlin and colleagues found that applying mechanical stress comparable to that generated during bronchoconstriction caused increased epithelial-release of ET-1 and 2 as well as TGF-β2 in normal PAEC-conditioned medium that stimulated lung fibroblast activation and increased synthesis of collagen [63]. These EMTU models showed how increased mechanical stress in asthmatic airways may cause epithelial damage that leads to increased growth-factor (ET-1/2, TGF-β2) release that can activate lung fibroblasts and cause excess ECM production.

In vitro co-culture models have shown that in the normal lung EMTU, the airway epithelium can also suppress fibroblast activation and excess ECM production [64]. Reeves and colleagues used airway epithelial ALI and lung fibroblast co-cultures to show that PAECs from normal individuals suppress the production of ECM proteins and profibrotic mediators such as collagen Iα1, collagen 3α1 and hyaluron synthase 2 by lung fibroblasts [21]. However, asthma-derived airway epithelial cells were less able to suppress the expression of these profibrotic molecules [21]. It was further shown that this defect may be due to a decreased expression of prostaglandin E2 synthase and an increased TGF-β2 release in the asthma-derived airway epithelium [21]. Further to this, Reeves and colleagues also used co-cultures to demonstrate that, compared to healthy controls, PAECs from asthmatic children were less able to suppress the expression of FMT markers such as α-SMA and tropomysin-I in lung fibroblasts [65]. This defect in epithelial regulation was again due to increased production of TGF-β2 emphasizing the importance of the TGF-β family in the aberrant communication that drives excess ECM production in the asthmatic EMTU [65]. 

The release of epithelial-IL-1α (and IL-1β) is also known to suppress the expression of ECM proteins [29]. In support of this, Osei et al., showed that stimulation of airway fibroblasts from asthmatics and control individuals cultured on collagen-1-coated plates with IL-1α/β caused a decreased expression of ECM proteins including collagen 1, fibronectin and periostin [31]. Further, IL-1 stimulation of airway fibroblasts embedded in a 3D free-floating low-tension collagen I gel inhibited the ability of airway fibroblasts to adequately contract collagen gels, leading to disorganization of collagen fibers [31]. This is worth noting since collagen fibers in asthmatic airways have been shown to be highly disorganized and fragmented [66]. IL-1 has also been shown to regulate TGF-β function, and vice versa, in epithelial-fibroblast co-culture models [29,67]. Osei et al. showed with a co-culture model that airway epithelial-IL-1α caused downregulation of TGF-β in lung fibroblasts [29]. Further, Mia and colleagues found epithelial-derived IL-1β inhibits TGF-β-dependent myofibroblast differentiation in control lung fibroblasts [67], while Zhang and Phan showed that TGF-β can inhibit IL-1-induced apoptosis of control lung myofibroblasts [68]. It will, therefore, be important in future work to assess the crosstalk of IL-1 and growth factors such as TGF-β, and how they may drive airway remodeling through ECM-regulation in asthma.

Taken together, these co-culture studies (summarized in Table 3) support the fact that epithelial release of growth factors (TGF-β1/2, TSP and ET-1), inflammatory mediators (PGE_2_) and loss of suppression of fibrotic changes (by TGF-β2)in the asthmatic EMTU, may cause increased production of ECM proteins from lung fibroblasts. The release of IL-1 from the airway epithelium may also be important for collagen fiber disorganization in the asthmatic airways. Understanding these subtle differences in how abnormal EMTU interactions contribute to ECM deposition in the airways shows the contributions of 3D in vitro co-culture models in elucidating the mechanisms of airway remodeling to aid in the search for new therapies in asthma.

## 5. Asthma EMTU Models and Therapeutic Studies

**Figure 3:**
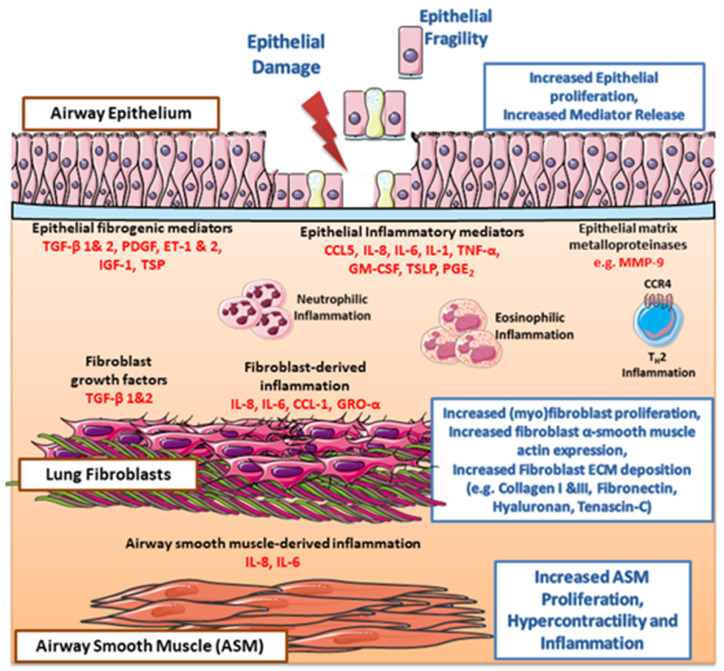
Epithelial-fibroblast interaction in the asthmatic epithelial-mesenchymal trophic unit. After damage to the airway epithelium, as a result of the exposure to allergens in asthmatic airways, the airway epithelium releases fibrogenic factors such as transforming growth factor (TGF)-β 1&2, platelet-derived growth factor (PDGF), endothelin (ET)-1&2, insulin-like growth factor (IGF)-1 and thrombospondin (TSP) that act on mesenchymal cells to induce increased cellular proliferation and ECM deposition (e.g., collagen 1 & III, fibronectin, hyaluronan, tenascin-C). The epithelium is also a source of a variety of inflammatory mediators including chemokine (C-C motif) ligand 5 (CCL5), interleukin (IL)-1, IL-8, IL-6, tumor necrosis factor (TNF)-α, granulocyte monocyte colony-stimulating factor (GM-CSF) and thymic stromal lymphopoietin (TSLP). These cytokines act on mesenchymal cells to cause the release of inflammatory mediators such as IL-8, IL-6, CCL1 and growth-regulated oncogene (GRO)-α. This inflammatory milieu stimulates neutrophilic, eosinophilic, and T_H_2 inflammation in different subtypes of asthma. The release of cytokines such as prostaglandin (PG)E_2_ and IL-1 also stimulates fibroblast ECM production and causes abnormal fibroblast ECM repair. Matrix metalloproteinases (MMP) such as MMP-9 are also released for the airway epithelium to stimulate mesenchymal cell proliferation and ECM production. In addition to this, mesenchymal cells produce growth factors such as TGF-β1 /2 that act on the airway epithelium to cause increased or decreased cell proliferation and remodeling in a feedback cycle that promotes airway remodeling and inflammation in the asthmatic EMTU.

The majority of preclinical therapeutic studies in asthma have been done in animal models. However, the anatomy, physiology, cell composition and inflammatory responses of the lungs in various animals are different from those of humans [69]. Co-culture models form part of the growing field of human in vitro preclinical models that employ primary cells from patients for the assessment of new therapeutic targets in human disease [70]. Many of the mediators (e.g., TGF-β1/2, ET-1, TSP-1, CCL5, IL-1, IL-8, IL-6, TNF-α, BNP; summarized in Figure 3), highlighted in this review to drive epithelial-mesenchymal cross-talk have previously been identified in asthma pathogenesis. However, with new insights into the complexity of their regulation using co-culture models, this may help to provide strategies to target these key mediators in asthma. As an example, the corticosteroids salbutamol and budesonide were shown to reduce TGF-β1 induced IL-8 and IL-6 release only in co-cultures of PAECs and ASM cells, while no effect was seen in monocultures [71]. The involvement of TGF-β signaling in inflammatory mediator release, as well as the expression of ECM proteins such as collagen in EMTU communication, points to its pleiotropic nature that requires further studies. To stimulate the synthesis of ECM proteins such as collagen, active TGF-β1/2, which is increased following allergen exposure and airway epithelial damage in asthma, binds to the TGF-β receptor (TGFBR)-II which phosphorylates TGFBR-1 that also phosphorylates the intracellular signal transducers Smad2 and 3 [72]. Phosphorylated Smad2 and 3 then bind and form a complex with coSmad4, which translocates into the nucleus to act as a transcription factor that regulates the expression of ECM proteins such as collagen [72]. Interestingly, it has been shown that high doses of an inflammatory cytokine such as IL-1 can activate Smads in the TGF-β pathway, while TGF-β can activate NF-κB inflammatory signaling, which points to a crosstalk between major regulatory cytokines that can be explored for future therapeutic studies [73].

Epithelial-hMSC co-culture studies have shown that cell-therapy using hMSCs may help abrogate airway epithelial-driven neutrophilia and T-cell inflammation in severe asthma models [35,36]. In line with this, after systemic injection of hMSCs in a mouse model of severe asthma developed by ovalbumin and polyI:C challenge, the cells were found to specifically accumulate near the airway epithelium and lung blood vessels. This hMSC accumulation in the lungs led to a marked reduction in neutrophils and macrophages in BAL fluid together with significant reductions in T_H_2-cytokines, IL-5, and CXCL15 [36]. These studies highlight as examples of how human in vitro co-culture models may be used to drive preclinical validation studies in the development of therapeutics for airway inflammation and remodeling in asthma.

## 6. The Future Outlook for In Vitro Co-Culture Models 

In addition to the co-culture models reviewed above, other in vitro model systems including lung-on-a-chip (LOAC), 3D bioprinting models, precision-cut lung slices (PCLS), and lung organoids hold future promise for understanding abnormal epithelial-mesenchymal crosstalk and assessing therapeutic targets in asthma. LOAC models are microfluidic biomimetic devices with separate microchannels that have continuous perfusion of air and fluids in which lung epithelial, mesenchymal, endothelial, and immune cells are cultured to mimic the in vivo environment [22]. As an example, the use of LOAC-models for therapeutic research has shown that the Janus kinase (JAK)-1, -2, and -3 pan-inhibitor Tofacitinib (Selleckchem) inhibits IL-13-induced asthmatic changes including goblet cell hyperplasia, increased GM-CSF, G-CSF and abnormal cilia movement [74]. Lung 3D bioprinting now enables the incorporation of lung cells with synthetic or ECM protein scaffolds [75]. A functional vascularized alveoli-like structure was recently printed using a synthetic hydrogel [76] and many studies are assessing the possibility of printing and implanting different lung structures to treat airway damage [77]. PCLS models involve ex vivo lung tissue cut into thin slices (300-500µM) which maintain structural integrity, cell-cell and cell-matrix interactions of the lung and are now extensively used in therapeutic studies [75]. For example, the corticosteroid budesonide, when used together with agonists (e.g., PGE_2_), enhances bronchodilation in human PCLS by increasing the release of cyclic adenosine monophosphate (cAMP) levels [78]. Lastly, lung organoids are developed by seeding progenitor and adult lung epithelial-mesenchymal cells in Matrigel, which allows them to self-aggregate into the airway or alveoli-like structures [79]. There are not many studies using asthma-derived cells in lung organoid-studies. However, they provide a useful platform to assess progenitor cell interactions with adult lung cells and their ability to repair damage in the asthmatic airways. The use of these complex 3D lung models offers the future potential of incorporation and assessment of all aspects of the asthmatic EMTU to aid in developing new treatments for airway inflammation and remodeling in asthma.

## 7. Conclusions

This review highlights the benefits of using 3D co-culture models to understand the complexity of epithelial-mesenchymal cross-talk in driving airway inflammation and remodeling in the setting of asthma. In summary, epithelial-derived mediators such as CCL5, IL-1, IL-8, IL-6 and TNF-α drive mesenchymal-mediated inflammation that promotes T_H_2, eosinophilic and neutrophilic inflammation in different asthma subtypes. The epithelial release of fibrogenic mediators such as TGF-β1/2, ET-, and TSP also has a distinct role in driving mesenchymal cell proliferation and ECM production (collagen I, III, HA, and tenascin-C) that are important features of airway remodeling in asthma. Further studies with rapidly developing complex multicellular human models are needed to understand how the balance in this communication might provide new insights for therapeutic studies in asthma. Of note, other cell-types in the EMTU, such as hMSCs, may also prove to be essential for therapy for inflammation and remodeling in asthma. The understanding of epithelial-mesenchymal interactions with its surrounding ECM and immune environment offers the potential for new therapeutic studies that could target the different pathogenic mechanisms in asthma. 

## Figures and Tables

**Table 1:** Summary of studies assessing airway inflammation in asthma with epithelial-mesenchymal trophic unit (EMTU) models.

In Vitro Model	Description	Mediator(s) Involved	Finding	Ref.
Co-culture	HRV-stimulated nonasthma-derived PAEC-ALIs cocultured with Asthma or nonasthma ASM cells	PAEC-release of CCL2, CCL5, CCL17, CXCL1, CXCL2, CXCL5, CXCL6, CXCL9, IL-1α, IL-6 and TNF-α	Specific release of CCL5 caused increased monocyte migration	[26]
Co-culture	Fibrocytes were cocultured with ASM cells	Increased activation of NF-κB-p65 and ERK1/2 in ASM cells	Increased release of IL-8 and IL-6 from ASM cells	[28]
Air-liquid interface PAEC-culture. Airway fibroblast culture on collagen-coated plates	Mediator-release assessed upon PAEC-differentiation. Cytokine stimulation of fibroblasts on collagen-coated plates	Increased release of IL-1α in asthma-derived PAECs when PAECs are in a monolayer before differentiation.	IL-1 stimulation cause IL-6, IL-8, TSLP and GM-CSF in airway fibroblasts	[31]
Conditioned medium (CM)	CM from BNP-treated BEAS-2B cells used to stimulate nonasthma and asthma ASM cells	Increased acetylcholine release from BNP-stimulated asthmatic ASM cells increase the expression of iNOS and MYPT1	Increased acetylcholine, iNOS and MYPT1 expression in ASM cells decrease histamine-induced hypercontractility of asthma-derived ASM cells	[34]
Co-culture	BEAS-2B cells pretreated with polyI:C or PBMCs stimulated with T-cell activator CD28 cocultured with hMSCs	PolyI:C treatment of BEAS-2B cells led to increased IL-8 release. CD28 caused IFN-γ and IL-4 released from PBMCs.	Increased IL-8 release from BEAS-2B cells as well as IFN-γ and IL-4 from PBMCs were inhibited upon co-culture with hMSCs	[35]
Co-culture	Hyperstretched PAECs co-cultured with hMSCs	Increased PAEC-release of IL-1α and IL-8 due to increased miR-155 expression that suppresses SHIP1 and cause JNK signaling	hMSC coculture with hyper-stretched PAECs causes IL-8 downregulation due to increased anti-inflammatory cytokine, IL-10 release.	[36]

**Table 2:** Summary of studies assessing cellular proliferation in asthma with EMTU models.

In Vitro Model	Description	Mediator(s) Involved	Finding	Ref.
3D-ECM Co-culture	16HBE14o cells treated with poly-L-arginine to mimic eosinophil granule cationic protein release in allergic asthmatics co-cultured on myofibroblasts embedded collagen 1 gels	Increased release of ET-1, PDGF, IGF-1, TGF-β2 from 16HBE cells	There was an increased myofibroblast proliferation in the 3D ECM co-culture	[19]
Co-culture	PAEC-ALIs subjected to repeated scrape wounds co-cultured with ASM cells	Increased PAEC release of MMP9, IL-8 and IL-6 after scrape wound.	MMP9 was the main mediator that caused ASM proliferation through ERK1 and MAPK activation	[42]
Conditioned medium (CM)	CM collected from compressed PAECs was used to stimulate ASM cells	PAEC compression caused increased ET-1 release	ET-1 release in PAEC-CM increased ASM proliferation and hypercontractility	[46]
3D-ECM Co-culture	PAEC-ALIs were co-cultured on collagen gels embedded with asthma and nonasthma-derived airway fibroblasts	Increased TGF-β1 release from asthma-derived airway fibroblasts led to an abnormal EGFR phosphorylation	Decreased PAEC proliferation rates in co-culture with mild asthma -fibroblast compared to nonasthmatics	[48]
Conditioned medium (CM)	CM from severe asthma/ nonasthma-derived airway fibroblasts was used to stimulate PAECs	Exosomes released from severe asthma-derived airway fibroblasts had a low expression of TGF-β2 and high expression of cytokines (IL-6, IL-8, CCL-1 and GRO-α).	Increased PAEC proliferation rates after stimulation with CM from severe asthma-derived airway fibroblasts due to low expression of TGF-β2 in exosomes	[50]

**Table 3:** Summary of studies assessing ECM homeostasis in asthma with EMTU models.

In Vitro Model	Description	Mediator(s) Involved	Finding	Ref.
3D-ECM Co-culture	Scrape-wounded PAECs co-cultured on lung fibroblasts embedded collagen 1 gels	Increased release of TGF-β2 from PAECs	Increased α-SMA, tenascin-C and fibrillar-collagen expression in lung fibroblasts	[54]
Co-culture	Scrape -wounded PAECs on human amnion chamber co-cultured on airway fibroblasts on plastic sheets	Scrape wound caused TGF-β1 and TSP release from PAECs	Myofibroblast induction and increased collagen I expression in airway fibroblasts	[55]
Co-culture	Nonasthmatic and asthmatic PAEC-ALIs co-cultured with normal lung fibroblasts	Increased PAEC- PGE_2_ production in asthmatic PAEC-ALIs	Increased collagen 1 and α-SMA expression in lung fibroblasts and fibroblast-to-myofibroblast transition	[58]
Co-culture	Nonasthmatic and asthmatic PAEC-ALIs co-cultured with normal lung fibroblasts	Increased hyaluronan synthase (HSA) 2 and 3 expressions in asthmatic PAEC-ALIs	Increased hyaluronan expression in lung fibroblasts	[59]
Co-culture & Conditioned Medium (CM)	PAECs isolated from ragweed-challenged asthmatics and nonasthmatics co-cultured with corresponding BAL fluid. Lung fibroblasts stimulated with CM from co-cultures	Increase TGF-β1 release from asthmatic-derived PAECs	Increased Collagen III expression in lung fibroblasts.	[60]
Co-culture	PAEC-ALIs subjected to varying degrees of mechanical strain co-cultured with lung fibroblasts	Increased MMP9 production and decreased TIMP1 expression in lung fibroblasts	Increased expression of fibronectin and collagen III in lung fibroblasts	[61]
Conditioned medium (CM)	Mechanical stress the same as bronchoconstriction applied to PAECs. CM from stressed PAECs used to stimulate lung fibroblasts	Increase in the PAEC release of ET-1, ET-2 and TGF-β2	Lung fibroblast activation and increased collagen synthesis	[63]
Co-culture	Nonasthmatic and asthmatic PAEC-ALIs co-cultured with normal lung fibroblasts	Decreased expression of PGE_2_ synthase, increased TGF-β2 release in the asthmatic-derived-PAECs	Asthmatic PAECs less able to suppress the expression of collagen Iα1, collagen 3α1 and HSA2 by lung fibroblasts	[21]
Co-culture	PAEC-ALIs from asthmatic children and normal individuals co-cultured with lung fibroblasts	Increased TGF-β2 production in asthmatic-derived PAECs	Asthmatic children PAECs less able to suppress the expression of FMT markers, α-SMA and tropomysin-I in lung fibroblasts	[65]
3D ECM free-floating collagen gel model. Airway fibroblast culture on collagen-coated plates	Cytokine stimulation of free-floating collagen 1-embedded asthmatic and nonasthmatic fibroblasts as well as fibroblasts cultured on collagen-coated plates	IL-1α, IL-1β and IL-33 stimulation of collagen 1 gel model and fibroblasts on collagen-coated plates	IL-1 inhibited airway fibroblast ability to contract collagen gels leading to collagen fiber disorganization and decreased expression of collagen 1, fibronectin and periostin	[31]
